# Approach for the Development of a Framework for the Identification of Activities of Daily Living Using Sensors in Mobile Devices

**DOI:** 10.3390/s18020640

**Published:** 2018-02-21

**Authors:** Ivan Miguel Pires, Nuno M. Garcia, Nuno Pombo, Francisco Flórez-Revuelta, Susanna Spinsante

**Affiliations:** 1 Instituto de Telecomunicações, Universidade da Beira Interior, 6201-001 Covilhã, Portugal; ngarcia@di.ubi.pt (N.M.G.); ngpombo@ubi.pt (N.P.); 2 Altranportugal, 1990-096 Lisbon, Portugal; 3 ALLab—Assisted Living Computing and Telecommunications Laboratory, Computing Science Department, Universidade da Beira Interior, 6201-001 Covilhã, Portugal; 4 ECATI, Universidade Lusófona de Humanidades e Tecnologias, 1749-024 Lisbon, Portugal; 5 Department of Computer Technology, Universidad de Alicante, 03690 Sant Vicent del Raspeig, Alicante, Spain; francisco.florez@ua.es; 6 Department of Information Engineering, Marche Polytechnic University, 60121 Ancona, Italy; s.spinsante@univpm.it

**Keywords:** Activities of Daily Living (ADL), environment, sensors, mobile devices, framework, data acquisition, data processing, data fusion, pattern recognition, machine learning

## Abstract

Sensors available on mobile devices allow the automatic identification of Activities of Daily Living (ADL). This paper describes an approach for the creation of a framework for the identification of ADL, taking into account several concepts, including data acquisition, data processing, data fusion, and pattern recognition. These concepts can be mapped onto different modules of the framework. The proposed framework should perform the identification of ADL without Internet connection, performing these tasks locally on the mobile device, taking in account the hardware and software limitations of these devices. The main purpose of this paper is to present a new approach for the creation of a framework for the recognition of ADL, analyzing the allowed sensors available in the mobile devices, and the existing methods available in the literature.

## 1. Introduction

Sensors embedded in off-the-shelf mobile devices, e.g., accelerometers, gyroscopes, magnetometers, microphones, and Global Positioning System (GPS) receivers [1], may be used in the development of algorithms for the recognition of Activities of Daily Living (ADL) [2] and the environments in which they are carried out. These algorithms are part of the development of a Personal Digital Life Coach (PDLC) [3]. According to [3], a PDLC “(…) will monitor our actions and activities, be able to recognize its user state of mind, and propose measures that not only will allow the user to achieve his/her stated goals, but also to act as an intermediate health and well-being agent between the user and his/her immediate care givers (…)”. This work is related to the development of ambient assisted living (AAL) systems, and, due to the increasing demands in our society, it is a field with high importance [4]. Due to recent advances in technology, there is an increasing number of research studies in this field for the monitoring of people with impairments and older people in a plethora of situations by using AAL technologies, including mobile devices and smart environments [5].

Multi-sensor data fusion technologies may be implemented with mobile devices, because they incorporate several sensors, such as motion sensors, magnetic/mechanical sensors, acoustic sensors, and location sensors [6], improving the accuracy of the recognition of several types of activities, e.g., walking, running, going downstairs, going upstairs, watching TV, and standing, and environments, e.g., bar, classroom, gym, library, kitchen, street, hall, living room, and bedroom. The selection of the activities and environments that will be included in the framework was based in the activities previously recognized with best accuracies, and, in the case of the environments, there are a lack of studies related to the environment recognition, taking into account some of the environments previously recognized and the most common environments [7]. The recognition of ADL may be performed with motion, magnetic/mechanical and location sensors, and the environments may be recognized with acoustic sensors. In order to improve the recognition of the ADL, the environment recognized may be fused with the other features extracted from the other sensors.

In accordance with previous works [6,8,9], the main motivation of this paper is to present the architecture of a framework for the recognition of ADL and their environments, which takes advantage of the use of a wide set of sensors available in a mobile device, also aiming at reducing the current complexity and constraints in the development of these systems. The test and validation of this framework is currently the subject of another step of this research plan [9], which includes the acquisition of a dataset that contains approximately 2.7 h of data collected from the accelerometer, gyroscope, magnetometer, microphone and GPS receiver, related to each activity and environment. During the collection phase, the data were acquired with the mobile device located in the front pocket of the trousers by 25 subjects aged between 16 and 60 years old and different lifestyles (10 mainly active and 15 mainly sedentary) and gender (10 female and 15 male). The activities performed and the environments frequented were labelled by the user. The subjects used their personal mobile phones with their applications running, where the mainly used device was a BQ Aquarius device [10].

The identification of ADL and environments using sensors has been studied during the last years, and several methods and frameworks [11,12,13,14,15,16] have been implemented using smartphones. However, this is a complex problem that should be separated into different stages, such as data acquisition, processing, and fusion; and artificial intelligence systems. The frameworks developed in previous studies are commonly only focused on some specific parts of the problem. For example, the Acquisition Cost-Aware QUery Adaptation (ACQUA) framework [17] has been designed for data acquisition and data processing, but it does not include all the steps needed for data processing.

There are no predefined standards for the creation of a framework for the recognition of the ADL [18,19,20], and the most implemented methods for the recognition of ADL are related to the use of motion sensors. However, there are methods and sensors that can be fused for the creation of a structured framework as a holistic approach to the identification of the ADL and environments presented in this paper.

Around the concept of sensors’ data fusion, the selection of the sensors to use is the first step for the creation of the framework, defining a method for the acquisition of the data, and, consequently, their processing. The processing of the data includes data cleaning, data imputation, and extraction of the features. Data segmentation techniques are not considered, as this study was designed for local execution on mobile devices and, due to the low memory and power processing restrictions of these devices, only a short sample of the sensors’ data can be used (initial research points to 5 s samples). This strategy makes it unsuitable to apply data segmentation techniques while still making it possible to deploy the framework in scarce resource devices. The final step in the proposed framework is the selection of the best features, and then the application of artificial intelligence techniques, i.e., the implementation of three types of Artificial Neural Networks (ANN), such as Multilayer Perceptron (MLP) with Backpropagation, Feedforward Neural Networks (FNN) with Backpropagation and Deep Neural Networks (DNN), in order to choose the best method for the accurate recognition of the ADL and the environments.

The remaining sections of this paper are organized as follows: Section 2 presents the state of the art in this topic, presenting a set of methods for each module/stage. Section 3 presents the framework for the identification of ADL using the sensors available in off-the-shelf mobile devices, the sensors and the methods that may be used. Section 4 presents a discussion and conclusions about the new approach proposed.

## 2. Related Work

Following previous research works related to the identification of ADL and the environment in which they are carried out, this Section reviews the state of the art on the sensors available on mobile devices (Section 2.1), data acquisition (Section 2.2), processing (Section 2.3), fusion (Section 2.4), artificial intelligence techniques (Section 2.5), and, finally, in Section 2.6, the methods to merge sensors’ data with users’ agenda.

### 2.1. Sensors

Sensors are small components that allow the acquisition of data when they are excited responding to stimuli, often external to the device. Available in many mobile devices, namely, in smartphones, sensors can be used to infer an ADL, and the combination of the data from multiple sensors can increase the efficiency of ADL identification, and environment recognition [9]. The number and types of sensors available on mobile devices is different for each mobile platform. In general, the sensors available in mobile devices are magnetic/mechanical sensors, environmental sensors, location sensors, motion sensors, imaging/video sensors, proximity sensors, acoustic sensors, optical sensors, and force sensors, being able to capture different types of signals, such as electrical, mechanical, acoustic and others [1,21].

Based on the classification presented in [6], sensors available on Android devices include microphones, accelerometers, gyroscopes, magnetometers, altimeters, humidity sensors, ambient light sensors, temperature sensors, GPS receivers, touch screens, microphones, and cameras [22,23]. In addition to platform-dependent restrictions in the use of sensors, the hardware differences between devices can influence the availability of specific sensors. Thus, the sensors available in most of the mobile devices, presented in Table 1, are the accelerometer, the gyroscope, the magnetometer, the GPS, the microphone, the touch screen, and the camera.

### 2.2. Data Acquisition

Data acquisition consists in the process of receiving the different types of data from the sensors available in the mobile devices. There are some possible problems that occur during the data acquisition process, including the influence of the unpredictable and uncontrolled external environment, the variability of the sampling rate of sensors, the number of tasks performed by the mobile device during the data acquisition, and the variability of the sensors chosen as input for a given developed framework [24]. Related to the variability of the position of the smartphone when carried by a user, to the best of the authors’ knowledge, there are no studies that solve this issue. As a standard method was not previously defined for the correct data acquisition and processing, and the sensors and capabilities of the mobile devices are different between manufacturers, the authors assumed that the results are nonetheless comparable.

In order to improve the data acquisition process, several frameworks have been developed, including Acquisition Cost-Aware QUery Adaptation (ACQUA) framework [17], Orchestrator framework [25], ErdOS framework [26], LittleRock prototype [27], Jigsaw continuous sensing engine [28], SociableSense framework [29], Continuous Hand Gestures (CHG) technique [30], and Barbie-Q (BBQ) approach [31].

The ACQUA framework allows to control the order of data acquisition, the correct segments of the data requested, the calibration of the data acquisition rates, the packet sizes and radio characteristics, the adaptation of the dynamic changes in query selective properties, and the support of multiple queries and heterogeneous time window semantics from all the sensors available in mobile devices, reducing the energy consumption of the real-time data acquisition [17].

The Orchestrator framework promotes the distributed execution of data acquisition using several mobile devices, and all devices execute a part of the data processing, avoiding to reduce the requirements related to the processing power and energy consumption [25].

The same purpose of Orchestrator framework is achieved from ErdOS framework and LittleRock prototype, distributing the data acquisition and processing processes by all resources available in the devices used, and reducing the energy needed to process the data collected from all sensors [26,27].

The Jigsaw continuous sensing engine implements a method to control the different sample rates, adapting the data acquisition and processing for the different capabilities of the sensors [28].

The SociableSense framework has a mechanism to adapt the different sample rates of all sensors used and it is a cloud-based framework, reducing the local data processing, but restricting the use of the framework to the availability of the Internet connection [29].

The authors of [30] implemented a CHG technique for the data acquisition with Windows Phone-based smartphones and low processing capabilities, capturing accelerometer and gyroscope data, storing the sensory data in the smartphone memory.

The BBQ framework applies a multi-dimensional Gaussian probability density function from all the sensors, inferring the order of the data acquisition with conditional probabilities [31].

The data acquisition process implemented in mobile devices may be performed without the use of frameworks, improving only the data processing according to the different resource capabilities. The authors of [32,33,34,35] implement the data acquisition process from accelerometer data in Apple iPhone and Android-based smartphones for the identification of several activities, including driving, walking, sitting, standing, running, and jumping activities. The authors of [36] implemented a Cursor Movement Algorithm to detect several activities, capturing the real-time data from the accelerometer and storing them into a local database in the mobile device.

Table 2 presents a summary of the data acquisition methods and their main characteristics for further implementation in the proposed new approach.

### 2.3. Data Processing

After the data acquisition process, the sensors’ data should be processed in order to prepare the data for the fusion from the chosen set of sensors, and, consequently, the application of the methods for ADL recognition. First, data processing should validate the integrity and quality of the data, and, then, applying data cleaning and/or data imputation techniques [37], in order to make this data available for the next stage in the processing pipeline of the framework. However, data processing depends on the environmental conditions, the types of sensors and data, the events of sensor failures, and the capabilities of the mobile devices [38]. Several techniques have been developed to reduce the memory and energy consumption of the data processing techniques. Other issues related to sensor drifting and generic noise are not specifically addressed in this paper, despite recognizing that sensors’ calibration and drift compensation may improve the outcomes of automatic recognition algorithms. Nevertheless, the application of data cleaning techniques mentioned in Section 2.3.1, and data imputation techniques mentioned in Section 2.3.2 may reduce the impact of drift and noise. Additionally, both the limited acquisition time used in the proposed framework and the fusion of data from different sensors, as discussed in [39], help in reducing the aforementioned effects. For each sensor data capture, we show that the use of only 5 s of sensors’ data is sufficient for the recognition of ADL and the environment. As a consequence the risk of failure in data acquisition or data corruption over such a short time may be assumed negligible.

The ACQUA framework is also used to optimize the data processing, by automated storage and retrieval system (ASRS) algorithms [17]. Other studies have presented approaches to adapt the data processing methods to the low capabilities of the mobile devices, processing the data after splitting or using methods with limited resources needed [24,40,41,42].

The use of data cleaning methods, presented in Section 2.3.1, is important to decrease the influence of the environmental conditions noise or systems failures. In order to improve the results, when the data acquisition fails, Section 2.3.2 presents the possible data imputation methods to correct the data acquired. However, these methods are not addressed by the proposed framework for the identification of ADL and their environments, assuming that the data acquired is sufficient for the extraction of several features from the, presenting the feature extraction methods and possible features to extract, in Section 2.3.3.

#### 2.3.1. Data Cleaning

Data cleaning consists in the identification of the incorrect values, removing outlier values and smoothing and filtering the invalid values obtained during the data acquisition process, commonly considered as noisy values [43,44,45]. Using data cleaning methods, the influence of the environmental conditions, the mobile device position, and system failures occurred during the data acquisition process is reduced. The efficiency of these methods depends on the type of data acquired and spatiotemporal characteristics of the data acquired.

The authors of [46] proposed a weighted moving average (WMA) algorithm that collects the sensors’ data and computes the weighted moving average, applying the WMA filter for the normalization and cleaning of the sensors’ data.

Three types of filters are used for the motion and magnetic/mechanical sensors: the low-pass filter (LPF), the high pass filter (HPF), and the KALMAN filter [47,48]. The WMA filter and the different types of Fourier transforms, such as Discrete Fourier Transform (DFT), Inverse Discrete Fourier Transform (IDFT), and Fast Fourier Transform (FFT) are also used to filter the acoustic data [49,50].

Table 3 presents a summary of the data cleaning methods related to the different types of sensors, discussed in Section 2.1. Concerning the implementation in the development of a framework for the identification of ADL and their environments, it can be seen that the LPF is commonly used in motion and magnetic sensors, the most used technique for acoustic sensors is the FFT and that the filtering techniques are not important for location, force and imaging sensors because of the nature of the values these sensors return.

#### 2.3.2. Data Imputation

During the data processing, the verification of the existence of faulty data is performed to flag that some values are missing in some instants of the acquired data time series. The data imputation methods are mainly used for motion sensors and magnetic/mechanical sensors. However, for the development of the new approach of the framework for the identification of ADL and their environments, the data imputation techniques were not considered, assuming that data acquired by the sensors is complete. Thus, in this section, the best methods for data imputation will be presented based on a literature review.

Faulty data may have different types that can be classified as Missing Completely At Random (MCAR), Missing At Random (MAR) and Missing Not At Random (MNAR) [51]. When the faulty data is randomly distributed during the time interval for the data acquisition, the classification of this data is MCAR. The other types of faulty data are MAR, verified when the faulty data is randomly distributed by different subsets of the data acquired, and MNAR, and verified when the faulty data is distributed by defined instants of the data acquisition.

The K-Nearest Neighbor (k-NN) method is one of the most used methods for data imputation of data acquired from motion, and magnetic/mechanical sensors [52,53,54,55]. The k-NN method has several variants that can be used for data imputation, such as MKNNimpute (K-nearest neighbor imputation method based on Mahalanobis distance), SKNNimpute (sequential K-nearest neighbor method-based imputation), and KNNimpute (K-nearest neighbor imputation) [52,53].

The clustering techniques are also used for the data imputation, including K-means clustering, K-means-based imputation, and fuzzy C-means clustering imputation [51,56,57], which are implement in the Imputation Tree (ITree) method presented in [51].

There are other methods related to data imputation, including multiple imputation [58], hot/cold imputation [59], maximum likelihood [60], Bayesian estimation [60], expectation maximization [54,61,62], discarding instances [18], pairwise deletion [18], unconditional mean imputation [18], conditional mean imputation [18], hot deck imputation [18], cold deck imputation [18], substitution method [18], linear regression [18], logistic regression [18], expectation-maximization (EM) algorithm [18], probabilistic neural networks [18], fuzzy min–max neural networks [18], general regression auto associative neural network [18], tree-based methods [18], multi-matrices factorization model (MMF) [63], mean imputation (MEI) [54,62], Multivariate Imputation by Chained Equations (MICE) [54,62], Fourier method [62], and Fourier and lagged k-NN combined system (FLk-NN) [54,62,64].

In general, these methods can be applied to data collection from motion and magnetic/mechanical sensors. Data imputation methods can also be applied to the acoustic data, being the more common the k-NN methods and singular value decomposition (SVD) algorithms [65].

As the data imputation methods should be able to impute the empty instances of the data acquired by motion and magnetic/mechanical sensors, the methods that are able to be used with this purpose are MEI, EM, MICE, and FLk-NN [54]. However, k-NN can be applied with the comparison between the history of the data acquisition that is similar to the data acquired in the stream with faulty values [54]. It emerges from the reviewed literature that data imputation may be avoided for acoustic and location sensors, because of the slow variability of their signals.

#### 2.3.3. Feature Extraction

The correct definition of the features extracted from the sensors’ data increases the accuracy of the identification of ADL and their environments. This definition depends on the types of sensors and the data acquired, but also on the purpose of their final use.

For the correct extraction of the features for the motion and magnetic/mechanical sensors’ data, the Euclidean norm for each instant of outputs from the sensors defined as magnitude of vector (MV). Thus, the features that should be extracted from the motion and magnetic/mechanical sensors are the mean for each axis [66,67,68,69], variance of MV [70,71], mean of MV [67,70,71,72,73,74,75], median of MV [70,74], maximum of MV [66,70,71,73], minimum of MV [66,70,71,73], standard deviation of MV [66,67,70,71,72,73,74,75], Root Mean Square (RMS) of MV [66,70], average of peak frequency (APF) of each axis [66], maximum of each axis [66,69,74], minimum of each axis [66,69,74], standard deviation of each axis [66,68,69], RMS of each axis [66], cross-axis signals correlation [66,67,69,73,76], Fast Fourier Transform (FFT) spectral energy [70,76], frequency domain entropy [76], FFT coefficients [70,73], logarithm of FFT [76], skewness of each axis [67], kurtosis of each axis [67], average absolute deviation of each axis [67], time between peaks [72], Interquartile range of MV [71,73], skewness of MV [71], kurtosis of MV [71], wavelet energy of MV [73], average of peak values [77], average of peak rising time [77], average of peak fall time [77], average time per sample [77], average time between peaks [77], slope for each axis [74], binned distribution for each axis [68], percentiles of MV [75], and zero crossing rate for each axis [69].

Related to the motion and magnetic/mechanical sensors’ data, the most used features are mean, standard deviation, maximum, minimum, median, correlation, variance, and FFT spectral energy of MV.

For the correct extraction of the features for the acoustic sensors’ data, the features that should be extracted are average [78], thresholding [78], minimum [78], maximum [78], distance [78], and MFCC (Mel-frequency cepstrum coefficients) [79,80].

For the location sensors, the feature that should be extracted is the distance travelled between a time interval, in order to identify ADL with high distance travelled. The distance between two points captured by a GPS receiver is the ellipsoidal distance, because the two points are acquired in the geodetic coordinate system, where the calculation of this distance is measured with the Vincenty formula [81,82,83].

Table 4 presents a summary of the features extracted for each type of sensors presented in the Section 2.1, for further implementation the in new approach for the development of a framework for the identification of ADL and their environments.

### 2.4. Data Fusion

After the extraction of the features, the data acquired from all sensors should be fused to improve the accuracy of the ADL identification and their environments in the new approach for the framework proposed in this study [11]. The data fusion methods implemented should be related with the final purpose of the framework presented in Section 2.6.

Based on the literature studies presented by several authors [12,20,84,85], the data fusion methods are grouped in four categories [12,84,85]. These are: probabilistic methods, statistical methods, knowledge base theory methods and evidence reasoning methods.

The probabilistic methods [12,20,84,85] include Bayesian analysis methods, maximum likelihood methods, state-space models, evidential reasoning, possibility theory, Kalman Filter [86,87], Particle filtering, k-Nearest Neighbor (k-NN), k-Means, optimal theory, uncertainty ellipsoids, Gaussian mixture model (GMM), weighted averages, and regularization.

The statistical methods [12,84,85] for data fusion include covariance intersection, cross-covariance, and other robust statistics. However, other statistical methods used for data fusion are dynamic time warping (DTW) [88], which measures the similarity between two temporal sequences, based on the raw data or the features extracted.

The knowledge base theory methods [12,20,84,85,89] for data fusion include Artificial Neural Networks (ANN), Support Vector Machines (SVM), Decision Trees, Deep Learning, Long Short Term Memory (LSTM) Recurrent Neural Networks (RNN), Fuzzy Logic, Topic models, and Genetics Algorithms.

The evidence reasoning methods [12,84,85] for data fusion include evidence theory, Bayesian network, Dempster-Shafer, and recursive operators.

Based on these categories of data fusion methods, several implementations have been performed and presented in several studies for the identification of a plethora of a real-life activities and environments. The Rao-Blackwellization unscented Kalman filter (RBUKF) [90] was implemented to fuse the data acquired from a compass, a gyroscope, and a GPS receiver. The Kalman filter was used to fuse the data acquired from the GPS receiver and the gyroscope in order to support a navigation system [91]. The Naïve Bayes classifier is used to fuse the data acquired from acoustic, accelerometer and GPS sensors to recognize different situations during daily life [92]. The Autoregressive-Correlated Gaussian Model was implemented in the KNOWME system [93]. Bayesian analysis and Kalman filter where used to data acquired from the several sensors available in mobile devices for the identification of the ADL [94]. The CHRONIOUS system implements several methods to recognize several ADL, such as Support Vector Machine (SVM), random forests, Artificial Neural Networks (ANN), decision trees, decision tables, and Naïve Bayes classifier, in order to fuse the data collection from several sensors available in mobile devices [95]. In [96], the authors used the empirical mode decomposition (EMD) applied to the inertial sensors available in a mobile device, including accelerometer, gyroscope, and magnetometer, for the identification of several ADL. The authors of [97] implements several methods for data fusion, including SVM, random forest, hidden Markov models (HMMs), conditional random fields (CRFs), Fisher kernel learning (FKL), and ANN for several sensors, such as Accelerometer, RFID, and Vital monitoring sensors for the correct identification of ADL.

Table 5 presents a summary of the data fusion methods that can be applied for each type of sensors presented in Section 2.1, for further implementation in a new approach for the development of a framework for the identification of ADL and their environments.

### 2.5. Identification of Activities of Daily Living

**Figure 1:**
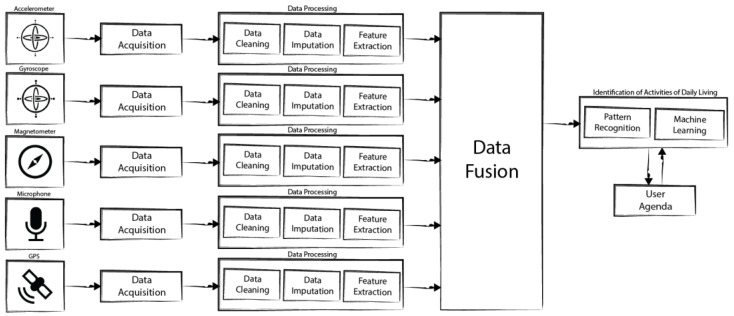
Schema for the framework for the recognition of Activities of Daily Living (ADL).

The definition of the methods for ADL identification represents the final module of the new proposed framework, presented in Figure 1. The identification of the ADL and their environments depends on the sensors’ data used, therefore, if a method uses the data acquired from motion and/or magnetic/mechanical sensors, it will probably be used to identify the ADL. If a method uses the data acquired from acoustic sensors, it will probably be used to identify the external environments. Finally, if the implemented method uses the location sensors, it is probably identifying activities with fast movement, e.g., driving, or it is probably trying to identify the place where the ADL is performed. In general, the identification of ADL is performed at the same time of the data fusion, because the methods use the same techniques.

The machine learning is a set of several techniques for artificial intelligence, including the techniques for the identification of ADL and their environments. The concept of machine learning will be presented in the Section 2.5.1. In Section 2.5.2, the pattern recognition methods are presented, which consists in a subset of the machine learning techniques.

#### 2.5.1. Machine Learning

Artificial Intelligence (AI) is one of the main areas for the development of computer science systems, and machine learning is composed by a subset of AI methods, where the computers have the ability to learn and perform some tasks, taking into account the external conditions of the system in order to change the execution of some methods for obtaining of better results [98].

Machine learning methods are based on the creation and implementation of algorithms for the recognition and prediction of several situations based on the data acquired, and these methods are commonly classified in four categories [99,100], such as Supervised learning, Unsupervised learning, Reinforcement learning, and Semi-supervised Learning and Active Learning.

Supervised learning methods are based on the automatic adjustment of the network parameters, comparing the actual network output with the desired output previously defined in the data acquired, where the error obtained is the mean squared error (MSE) [100]. The input data involved in the supervised leaning should be labeled, in order to perform the comparisons.

Unsupervised learning methods consist on the correction of the results obtained based on the input data, attempting to obtain the signification patterns or features in the unlabeled input data, automatically learning with intuitive primitives like neural competition and cooperation [100].

Reinforcement learning methods are similar to supervised learning methods, but the exact desired output is *a priori* unknown [100]. Thus, these methods are learning based on the feedback provided during the execution of the algorithm by an artificial agent in order to maximize the total expected reward [100].

Semi-supervised Learning and Active Learning methods are methods that should be applied to datasets with a large collection of unlabeled input data and a few labeled examples to generalize the results and performance of the method, based on assumptions related to the probability of occurrence of some output.

For the development of a new approach for the development of a framework for the identification of ADL and their environments, the machine learning may be used, as it can be adapted to bioinformatics and human-related systems [101,102,103,104]. Pattern recognition methods, described in Section 2.5.2, consist on a subset of machine learning methods for the recognition of patterns [105], which are very useful in the development of the framework for the identification of ADL and their environments.

#### 2.5.2. Pattern Recognition

The use of pattern recognition methods is the final part of research for the creation of a new approach for a framework for the identification of ADL and their environments. Several sensors, presented in Section 2.1, may be used with pattern recognition methods, which should be applied to the features extracted from the input data.

The methods implemented during the pattern recognition step are similar to the methods implemented for the data fusion, presented in Section 2.4. As reported early in this paper, the data fusion and pattern recognition may be confused, and the pattern recognition is performed at the same time of the data fusion. The categorization of the methods is similar to the methods applied for data fusion, and they are separated in four categories [12,84,85], these are the probabilistic methods, the statistical methods, the knowledge base theory methods and the evidence reasoning methods.

Several ADL may be recognized with pattern recognition methods, as example for the recognition of standing, and walking activities may be used ANN [106]. Several authors [13,14,15,16,66,67,68,69,71,72,73,74,75,76,89,107,108,109,110,111,112,113,114,115,116,117,118,119,120] proposed the use of the ANN, probabilistic neural networks (PNN), deep neural networks (DNN), Long Short Term Memory (LSTM) Recurrent Neural Networks (RNN), SVM, Random Forest, Bayesian Network, Sequential Minimal Optimization (SMO), Logistic Regression, Naïve Bayes, C4.5 Decision Tree, Logistic Model Trees (LMT), J48 Decision tree, K-Nearest Neighbor (KNN), and Simple Logistic Logit Boost methods for the recognition of walking, running, jogging, jumping, dancing, driving, cycling, sitting, standing, lying, walking on stairs, going up on an escalator, laying down, walking on a ramp activities, cleaning, cooking, medication, sweeping, washing hands, and watering plants.

The Hidden Markov Model (HMM) and their variants are also a pattern recognition implemented in several studies related with the identification of ADL and their environments, such as the Hidden Markov Model (HMM) [71], the Hidden Markov Model Ensemble (HMME) [121], the Sliding-Window-based Hidden Markov Model (SW-HMM) [113]. The ADLs commonly identified by the HMM method are walking, walking on stairs, standing, running, sitting, and laying.

Table 6 presents a summary of the pattern recognition methods that can be applied for each type of sensors presented in Section 2.1, for further implementation in the proposed approach for the identification of ADL and their environments. As shown in the Table, the HMM method is commonly used for the recognition of walking, walking on stairs, standing, running, sitting and laying activities, whereas the SVM, ANN and their variants, HMM and Random Forest methods, are useful for the recognition of complex activities (e.g., cleaning, cooking, medication, sweeping, washing hands and watering plants). However, all of the described methods in this study may be used for the recognition of simple activities (e.g., walking, running, jogging, jumping, dancing, driving, cycling, sitting, standing, lying, walking on stairs, going up on an escalator, laying down and walking on a ramp) with reliable accuracy.

### 2.6. Relation between the Identification of Activities of Daily Living and User Agenda

After the identification of the ADL and their environments with machine learning methods, the results obtained should be compared with the users’ agenda for the validation of the scheduled activities performed during the daily life. By comparing the identified ADL with the user’s agenda, it will be possible to monitor the lifestyle [122] and provide feedback regarding planned activities and executed activities. However, the inputs from agenda can also be used to validate the accuracy of the framework developed [123].

## 3. Methods and Expected Results

The new approach proposed for the creation of the framework for the identification of ADL (Figure 1) is based on [6,8,9], and it is composed by several stages. They are: the selection of the sensors, the data and processing, including data cleaning, imputation, and feature extraction, data fusion, the identification of ADL with artificial intelligence, including pattern recognition, and other machine learning techniques, and, at the end, the combination of the results obtained with the data available in the users’ agenda.

In order to create a new approach for a framework for the identification of ADL and their environments, the architecture, presented in Figure 1, and set of methods presented in Section 2 are proposed for obtaining results with reliable accuracy.

Following the list of sensors available in off-the-shelf mobile devices, presented in Section 2.1, the sensors that will be used in the framework should be dynamically selected, according to the sensors available in the mobile device. Thus, the types of sensors selected to use in the framework will be motion sensors, magnetic/mechanical sensors, acoustic sensors, and location sensors. The accelerometer is available in all mobile devices, but the gyroscope is only available on some devices, therefore, to cover the execution of the framework in all devices, two different methods should be implemented, one considering the data from the accelerometer and the gyroscope, and another considering only the data from the accelerometer. The magnetometer is only available on some devices, therefore this sensor should be managed similarly. Related to the acoustic sensors, the microphone is available in all mobile devices. As to the location sensors, the GPS is available in most of the mobile devices and its data should be used in the framework whenever possible.

The data acquisition methods are not directly related to the development of the framework, because the different manufacturers of the mobile operating systems have different methodologies to acquire the different types of sensors’ data. Thus, the data acquisition methods, presented in Section 2.2, should take in account the limitations of the mobile devices. Based on previous research studies and preliminary experiments, acquiring only 5 s of data from the selected sensors every 5 min is sufficient for the identification of the ADL and environments.

Following the creation of the new approach for a framework for the identification of ADL and their environments, the selection of data processing methods, presented in Section 2.3, should contain the data cleaning, data imputation, and feature extraction methods.

The data cleaning methods adapted for the framework depends on the types of sensors. On the one hand, for the accelerometer, gyroscope, and magnetometer sensors, the data cleaning method that should be applied is a low pass filter to remove the noise and the value of the gravity acquired during the data acquisition process. On the other hand, for the acoustic sensors, the data cleaning method that should be applied is the FFT in order to extract the frequencies of the audio. As the location sensors return values that are in nature already a result (e.g., GPS coordinates), data cleaning methods are not significant. Nevertheless, and as future work, it may be necessary to implement algorithms that increase the accuracy of these sensors as to better contribute to a quality data fusion process.

The data imputation methods is not important to implement in the development of a new approach for a framework for the identification of ADL and their environments, assuming that the data acquired from all sensors is always filled.

Related to the feature extraction, the features needed to recognize the ADL and their environments should be selected based on the type of sensors and on the selected features already reported in the literature and presented in Section 2.3.3. Firstly, the features selected for the accelerometer, gyroscope, and magnetometer sensors are the five greater distances between the maximum peaks, the average of the maximum peaks, the standard deviation of the maximum peaks, the variance of the maximum peaks, the median of the maximum peaks, the standard deviation of the raw signal, the average of the raw signal, the maximum value of the raw signal, the minimum value of the raw signal, the variance of the of the raw signal, and the median of the raw signal. Secondly, the features selected for the microphone are the standard deviation of the raw signal, the average of the raw signal, the maximum value of the raw signal, the minimum value of the raw signal, the variance of the of the raw signal, the median of the raw signal, and 26 MFCC coefficients. Finally, the features selected for the GPS receiver are the distance travelled during the acquisition time.

**Figure 2:**
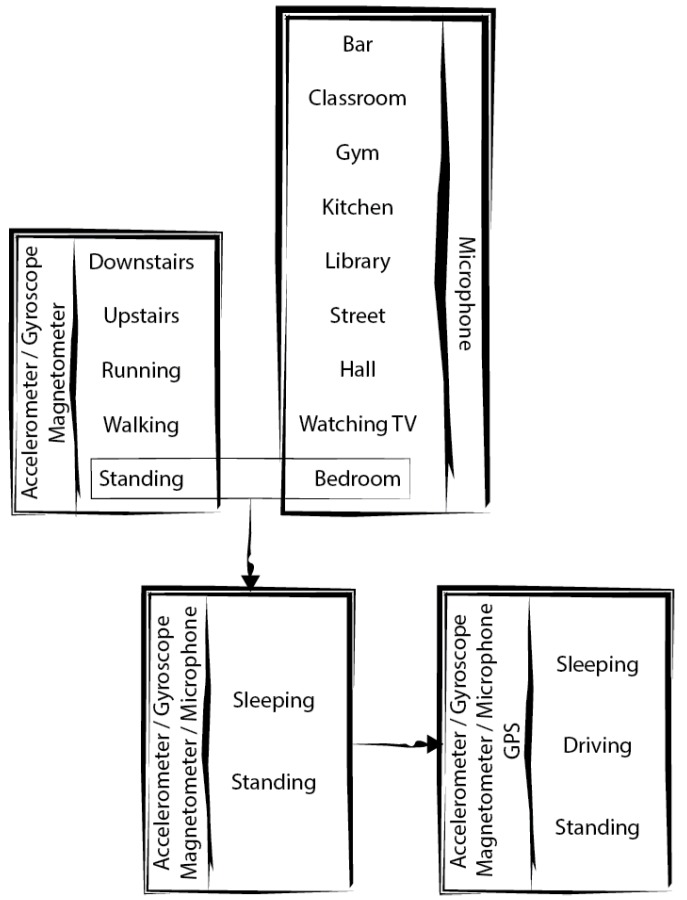
Sensors used for the recognition of Activities of Daily Living (ADL) and environments for each phase of development.

Before the presentation of the data fusion and pattern recognition methods that should be used for in the framework, the ADL and environments to recognize should be defined. This process should be executed with several sensors, that will be combined as presented in the Figure 2 and Table 7, being these the necessary stages:

1. Firstly, the ADL are recognized with motion and magnetic/mechanical sensors;
2. Secondly, the identification of the environments is performed with acoustic sensors;
3. Finally, there are two options, being these:
  ○ The identification of standing activities with the fusion of the data acquired from motion and magnetic/mechanical sensors, and the environment recognized, where the number of ADL recognized depends on the number of sensors available;
  ○ The identification of standing activities with the fusion of the data acquired from motion, magnetic/mechanical and location sensors, and the environment recognized, where the number of ADL recognized depends on the number of sensors available.

In identifying the environments, what is intended is to identify the associated activity, i.e., the sound generated in a classroom is not only the sound of the room itself, but rather the sound of a class who is having a lesson in a classroom. This is to say that an environment is to be considered as a place where some activity occurs in a given time of the day or the week, so there will be the need to consider different types of “Street” environments as they will have different audio signatures at different times of the day or week and of course, in different streets. All the proposed environments shown in Figure 2 are expected to be plural.

Firstly, the ADL to be identified with the framework will be going downstairs, going upstairs, running, walking, and standing, because they are part of the most recognized ADL in previous studies with reliable accuracy [7]. Secondly, the proposed environments to identify with the framework will be bar, classroom, gym, kitchen, library, street, hall, watching TV, and bedroom, because the existence of previous studies related to the recognition of environments is very limited, the proposed framework will take in account the most common environments and some of the environments previously recognized [7]. Thirdly, the proposed ADL to distinct with the framework will be sleeping, and standing, because the ADL may be confused as standing ADL and the inclusion of the environment recognized as an input for the classification method will help in the accurate recognition of these ADL. Finally, the proposed ADL to distinct with the framework are sleeping, standing, and driving, because the driving may also confused as standing ADL and, in order to accurately distinct these ADL, the environment recognized and the features extracted from the GPS receiver should be included. As the data for the creation of the methods for the recognition of ADL and environments was acquired will several conditions and different people, the generated method with ANN will be generic and the calibration of sensor is not needed.

Based on the list of data fusion methods and pattern recognition methods, defined in Section 2.4 and Section 2.5, the method selected for the implementation in the new approach for a framework for the identification of ADL and their environments will be based in ANN methods, because, based on the literature, it is one of the methods that reports the best accuracies. However, the selection of the best type of ANN will be done with the comparison of the results obtained with three types of ANN selected. The types of ANN that will be tested to the acquired data are:

- MLP with Backpropagation;
- FNN with Backpropagation;
- DNN.

Regarding the data acquired from GPS receiver, it can be useful to increase the accuracy of the identification of the ADL and their environments, but it can also be used for the identification of the location where the ADL are executed, in order to improve the comparison with the users’ agenda presented in Section 2.6. 

## 4. Discussion and Conclusions

This paper presents the architecture of a new approach for a framework for the identification of ADL and their environments, using methods with a reported good accuracy. The development of the new approach for the development of a framework for the identification of ADL and their environments, based on the system presented in [6,8,9], is one of the steps for the creation of a personal digital life coach [3] using mobile devices.

The framework will be composed by several modules several, such as data acquisition, data processing, data fusion, and a module to implement artificial intelligence techniques for the identification of the ADL and their environments.

The sensors used in the framework will be accelerometer, gyroscope, magnetometer, microphone, and GPS receiver, in order to recognize several ADL, including going downstairs, going upstairs, running, walking, standing, sleeping, and driving, and their environments, including bar, classroom, gym, kitchen, library, street, hall, watching TV, and bedroom.

The sensors’ data should be acquired and, before the extraction of the features of the sensors’ data, filters such as low pass filter and FFT, should be applied. Afterwards, the data fusion and pattern recognition methods should be applied for the recognition of ADL and environments.

This paper consists on a conceptual definition of the framework for the recognition of the ADL and their environments, proposing three possible methods for this purpose, based on the use of the ANN methods. In order to define the best method, the future implementation of the proposed methods will compare the differences between them, including the accuracy, performance, and adaptability for the development of a local processing framework for mobile devices. It will include the acquisition of a large set of sensors’ data related to the ADL and environments proposed for the creation of training and testing sets and further validation of the developed methods. Additionally, and also as future work, the framework will allow each user to validate the ADL identified by the framework when this is not the real performed activity.

Due to the inexistence of previous studies that review the use of all sensors available in current off-the-shelf mobile devices, our proposed framework is a function of the number of sensors available in the mobile device used, proving a reliable feedback in almost real-time.

## Figures and Tables

**Table 1:** List of sensors available in mobile devices.

Categories:	Sensors:	Availability
Motion sensors	Accelerometer Gyroscope	Always present Present in most models
Magnetic/mechanical sensors	Magnetometer	Present in most models
Location sensors	GPS	Always present
Acoustic sensors	Microphone	Always present
Force sensors	Touch screen	Always present
Imaging/video sensors	Camera	Always present

**Table 2:** Summary of the data acquisition methods.

Methods:	Advantages:
ACQUA framework [17]	Controls of the order of the data acquisition; Controls the correct segments of the data requested; Controls the calibration of the data acquisition rates; Controls the packet sizes and radio characteristics; Controls the adaptation of the dynamic changes in query selective properties; Controls the support of multiple queries and heterogeneous time window semantics; Adapted for low processing, memory, and energy capabilities.
Orchestrator framework [25]	Distributed execution of the data acquisition using several mobile devices; Adapted for low processing, memory, and energy capabilities.
ErdOS framework [26]	Distributed execution of the data acquisition using several mobile devices; Adapted for low processing, memory, and energy capabilities.
LittleRock prototype [27]	Adapted for low processing, memory, and energy capabilities.
Jigsaw continuous sensing engine [28]	Controls the different sample rates; Adapted for low processing, memory, and energy capabilities.
SociableSense framework [29]	Cloud-based framework; Needs a constant Internet connection; Adapted for low processing, memory, and energy capabilities.
CHG technique [30]	Stores the sensory data in the smartphone memory; Adapted for low processing, and energy capabilities.
BBQ framework [31]	Uses a multi-dimensional Gaussian probability density function from all sensors; Adapted for low processing, memory, and energy capabilities.
Cursor movement algorithm [36]	Stores the sensory data in the smartphone memory; Adapted for low processing, and energy capabilities.
No framework	Adapted for low processing, memory, and energy capabilities.

**Table 3:** Relation between the types of sensors and the data cleaning techniques allowed.

Types of Sensors:	Data Cleaning Techniques:
Motion sensors; Magnetic/mechanical sensors.	Low-Pass Filter; High-Pass Filter; KALMAN Filter; Weighted moving average (WMA) algorithm; Moving average filter.
Location sensors	The data cleaning technique is not important for this type of data acquired.
Acoustic sensors	Moving average filter; Discrete Fourier Transform (DFT); Inverse Discrete Fourier Transform (IDFT); Fast Fourier Transform (FFT).
Force sensors Imaging/video sensors	The data cleaning technique is not important for this type of data acquired.

**Table 4:** Relation between sensors and extracted features.

Types of Sensors:	Features:
Motion sensors; Magnetic/mechanical sensors.	Mean [67,70,71,72,73,74,75], average of peak frequency (APF) [66], maximum [66,70,71,73], minimum [66,70,71,73], standard deviation [66,67,70,71,72,73,74,75], Root Mean Square (RMS) [66,70], cross-axis signals correlation [66,67,69,73,76], skewness [67], kurtosis [67], average absolute deviation [67], slope [74], binned distribution [68], and zero crossing rate for each axis [69]; Mean [67,70,71,72,73,74,75], median [70,74], variance [70,71], maximum [66,70,71,73], minimum [66,70,71,73], standard deviation [66,67,70,71,72,73,74,75], Root Mean Square (RMS) [66,70], Fast Fourier Transform (FFT) spectral energy [70,76], frequency domain entropy [76], FFT coefficients [70,73], logarithm of FFT [76], Interquartile range [71,73], skewness [67], kurtosis [67], wavelet energy [73], and percentiles of MV [75]; Time between peaks [72], average of peak values [77], average of peak rising time [77], average of peak fall time [77], average time between peaks [77].
Location sensors	Distance between two points.
Acoustic sensors	Average [78], Thresholding [78], Minimum [78], Maximum [78], Distance [78], MFCC (Mel-frequency cepstrum coefficients) [79,80].
Force sensors; Imaging/video sensors.	These sensors are not useful for the development of the framework for the Identification of ADL and their environments.

**Table 5:** Relation between the different types of sensors and some data fusion methods.

Types of sensors:	Data fusion methods:
Motion sensors; Magnetic/mechanical sensors; Location sensors; Acoustic sensors.	Autoregressive-Correlated Gaussian Model; Fuzzy Logic; Dempster-Shafer; Evidence Theory; Recursive Operators; Support Vector Machine (SVM); Random Forests; Artificial Neural Networks (ANN); Decision Trees; Naïve Bayes classifier; Bayesian analysis; Kalman Filter; k-Nearest Neighbor (k-NN); Least squares-based estimation methods; Optimal Theory; Long Short Term Memory (LSTM) Recurrent Neural Networks (RNN); Uncertainty Ellipsoids.
Force sensors; Imaging/video sensors.	These sensors are not useful for the development of the framework for the Identification of ADL and their environments.

**Table 6:** Relation between the different types of sensors and some pattern recognition methods.

Types of Sensors:	Pattern Recognition Methods:	ADL Recognized:
Motion sensors; Magnetic/mechanical sensors; Location sensors; Acoustic sensors.	Support Vector Machines (SVM); Decision trees (J48, C4.5); Artificial Neural Networks (ANN); Probabilistic Neural Networks (PNN); Deep Neural Networks (DNN); Long Short Term Memory (LSTM) Recurrent Neural Networks (RNN); k-Nearest Neighbour (KNN); Naïve Bayes; Random Forest; Logistic Regression; Bayesian network; Sequential minimal optimization (SMO); Logistic Model Trees (LMT); Simple Logistic Logit Boost.	Walking; running; jogging; jumping; dancing; driving, cycling; sitting; standing; lying; walking on stairs; going up on an escalator; laying down; walking on a ramp.
	Support Vector Machines (SVM); Artificial Neural Networks (ANN); Probabilistic Neural Networks (PNN); Deep Neural Networks (DNN); Long Short Term Memory (LSTM) Recurrent Neural Networks (RNN); Hidden Markov model (HMM); Random Forest.	Cleaning; cooking; medication; sweeping; washing hands; watering plants.
	Hidden Markov model (HMM).	Walking; walking on stairs; standing; running; sitting; laying.
Force sensors; Imaging/video sensors.	These sensors are not useful for the development of the framework for the Identification of ADL and their environments.	

**Table 7:** Sensors, Activities of Daily Living (ADL), and environments for recognition with the framework proposed.

		Accelerometer	Gyroscope	Magnetometer	Microphone	GPS
Activities	Going Downstairs	✓	✓	✓		
	Going Upstairs	✓	✓	✓		
	Running	✓	✓	✓		
	Walking	✓	✓	✓		
	Standing	✓	✓	✓	✓	✓
	Sleeping	✓	✓	✓	✓	✓
	Driving	✓	✓	✓	✓	✓
Environments	Bar				✓	
	Classroom				✓	
	Gym				✓	
	Library				✓	
	Kitchen				✓	
	Street				✓	
	Hall				✓	
	Watching tv				✓	
	Bedroom				✓	
